# Telehealth for Expanding the Reach of Early Autism Training to Parents

**DOI:** 10.1155/2012/121878

**Published:** 2012-11-22

**Authors:** Laurie A. Vismara, Gregory S. Young, Sally J. Rogers

**Affiliations:** Department of Psychiatry and Behavioral Sciences, Medical Investigation of Neurodevelopmental Disorders (MIND) Institute, University of California, Davis 2825 50th Street, Sacramento, CA 95817, USA

## Abstract

Although there is consensus that parents should be involved in interventions designed for young children with autism spectrum disorder (ASD), parent participation alone does not ensure consistent, generalized gains in children's development. Barriers such as costly intervention, time-intensive sessions, and family life may prevent parents from using the intervention at home. Telehealth integrates communication technologies to provide health-related services at a distance. A 12 one-hour per week parent intervention program was tested using telehealth delivery with nine families with ASD. The goal was to examine its feasibility and acceptance for promoting child learning throughout families' daily play and caretaking interactions at home. Parents became skilled at using teachable moments to promote children's spontaneous language and imitation skills and were pleased with the support and ease of telehealth learning. Preliminary results suggest the potential of technology for helping parents understand and use early intervention practices more often in their daily interactions with children.

## 1. Introduction

Parents are their children's first and most natural teacher and thus are in a unique position to influence their early years of development [1, 2]. Helping parents become proficient and long-lasting agents of change necessitates specialized training and support of their skill use throughout their daily routines. While smaller studies have noted positive behavioral gains for children with autism spectrum disorder (ASD) when parents are taught to implement interventions at home [3–6], recent randomized controlled trials involving large sample sizes have failed to demonstrate expected main effects on child outcomes from parent-delivered interventions (Carter et al., 2011; [7–9]). This does not appear to be due to ineffective intervention strategies, as evidenced by significant gains in children's social-communicative behavior when the same intervention procedures are used by professionals [10, 11]. It also does not appear to be due to parents' inability to learn and demonstrate the skills in supporting children's communication [2, 6, 12], play and joint attention [13], imitation [4], and shared engagement [10, 13, 14].

Instead, the lack of significant outcome differences for parent-delivered intervention may require that parents receive more instruction and practice with the content inside real-life moments and interactions with children [8]. However, working with families where the most interaction is likely to occur (i.e., their homes) may be difficult to arrange because of daily demands, family schedules (e.g., siblings, work, family life), and waiting lists for in-home intervention [15]. As a result, families may not accurately develop the skill necessary to help their children learn or have extreme difficulty obtaining the intervention, itself [2]. The goal of the current study was to examine a novel approach for delivering parent-implemented intervention in families' homes and supporting parent-child learning. 

Telehealth uses communication technologies to deliver specialized services in real time over a geographical distance [16, 17]. It can be accessed at any time of day, in any location with basic, inexpensive equipment, and uses interactive, personalized features to communicate and share information [18]. Telehealth such as computerized software programs, videoconferencing, and virtual 3D interactive programs has been used to teach various communicative, social, emotional, and academic skills to older children and adolescents with ASD (see [19, 20] for reviews). However, the majority of this training has occurred in simulated environments [21], classroom settings [22, 23] or through online distance learning programs with professionals [24–26] rather than in families' homes. Those telehealth programs that do offer parenting resources have primarily focused on behavior management and general adaptive parenting techniques aimed at helping high risk parents and those with behaviorally challenged children rather than families affected by autism (e.g., [18, 27–30]). 

To date, only a few published studies have examined the efficacy of telehealth intervention for parents of children with ASD. Baharav and Reiser [31] found that online video-conferencing sessions in two families' homes, allowing therapists to provide live feedback and coaching to parents implementing speech and language therapy, supported the child gains that occurred in traditional therapy settings over a 6-week period. Parents described the telehealth sessions at home as comparable to clinic-delivered sessions and felt comfortable using the technology to communicate with therapists. Similarly, Nefdt et al. (2010) [32] found that a group of parents of children with ASD (*n* = 13) developed greater confidence and intervention skill, and provided more language opportunities for their children, using an interactive, self-guided DVD than did parents in the control group (*n* = 14). Jang et al. (2012) [33] found that parents' knowledge of applied behavior analysis procedures increased after participating in an e-Learning or web-based training module compared to a wait-list control group. Additional research is necessary to examine parents' actual implementation of the targeted intervention; however, the findings to date suggest that telehealth may make evidence-based intervention more available to families with ASD without intensive and potentially costly involvement of professionals.

The current study used telehealth to deliver a parent intervention curriculum over the course of 12 one-hour per-week sessions to nine parents and toddler-aged children with ASD. Previously, the parent intervention model had been tested in a center-based approach with positive parent-child gains compared to baseline levels of performance and sustained across a brief follow-up period [6]. The current study is an attempt to move those clinic-based procedures into the parents' home, using video-conferencing and a DVD learning module to deliver similar materials and coaching practices. The study predicted that telehealth delivery of a parent intervention would be feasible to carry out with families in their homes with increased parent fidelity, child learning, and parental acceptance of this unique approach to learning.

## 2. Method

### 2.1. Participants

Selection criteria included the following: (a) children no older than 36 months of age; (b) the same parent in attendance for all video-conferencing sessions in order to monitor performance across outcome measures; (c) no intensive treatment as defined by 10 or more hours of in-home or center-based intervention during the 12 weeks of intervention; (d) a diagnosis of autism spectrum disorder completed by a licensed professional in the families' community using the Autism Diagnostic Observation Schedule (ADOS [34]) and parental consent to share results; and (e) Internet availability from their home throughout the duration of the study (although a laptop and web-camera were available should families not have this equipment). Nine families (seven mothers and two fathers) self-referred to the study based upon reading about the ESDM from research articles, the center's website, and/or the published manual [35] and were accepted into the study on a first come, first served basis. One family dropped out of the study after completing only four intervention sessions due to enrollment in an intensive, center-based program; whereas another family completed all 12 intervention sessions, but terminated during follow-up because of serious health issues. Both families' data were included in analyses when available. Thus, eight out of the nine families originally enrolled completed intervention; whereas seven families completed follow-up. 

Families represented middle-class status, all with established Internet connections from their homes and a laptop or computer and web-camera already available for participation. All but one parent was married and were Caucasian with the exception of one Hispanic parent. Out of the nine children, only one was female, and all families lived in various states including California, North Carolina, Arkansas, Texas, and Pennsylvania. All families had very little access to additional early intervention services, generally involving only one hour each of speech and occupational therapy per week. One child had been receiving up to 13 hours per-week of intervention delivered in the family's home for three months prior to starting the study. His parents and in-home therapist were extremely concerned about the child's lack of progress and had ceased participation with the prior intervention at the start of the study. Children's average age at the beginning of treatment was 28.89 months (SD = 7.64, range 16 to 38 months). Six children were diagnosed with autism and 3 were diagnosed with PDD-NOS as assessed by licensed professionals in the families' community. These evaluations also included standardized testing using the Mullen Scales of Early Learning [36], ADOS [34], Vineland Adaptive Behavior Scales 2nd Edition ([37]; see Table 1 for preintervention descriptive statistics). All research activities were approved by the university's Institutional Review Board and families were consented through the synchronous, two-way video conferencing program prior to their participation in the study. 

### 2.2. Setting and Materials

 All sessions were conducted on an Internet-based, password-protected video-conferencing program using computers or laptops and web-cameras to allow the therapist and parents to see, hear, and communicate with one another in real time. The therapist accessed the program from an office computer and web-camera, whereas all parents had their own laptops and web-cameras to access the program from their home although equipment was available to those in need. Prior to weekly intervention sessions, the therapist and parent strategized on locations in different rooms (e.g., family room, kitchen, bedroom) to place the laptop that would allow unobstructed views of parent-child activities. Most often, parents placed their laptop in the center of the floor, on a table, or chair and preferred to be in their family room, kitchen, or bedroom. Some parents solicited the help of their spouse, family member, speech or occupational therapist, or babysitter to operate the camera; otherwise these preplanned locations helped maintain clear visibility and audible communication between parents and therapist. Parents used their child's preferred toys, snack or meal items, and/or physical games (e.g., chase, airplane, tickle) as motivating materials to engage their child in activities.

### 2.3. Therapist Training

 Parent intervention sessions were provided to all families by the first author who had received extensive training and supervision in the ESDM by one of the model's developers and had codeveloped and piloted the ESDM parent coaching curriculum in prior research before conducting this study. The therapist followed a coaching protocol to ensure that the interpersonal, collaborative coaching practices of the ESDM (adapted from [38]) remained intact and were used consistently when working remotely with families. The protocol outlined the session structure (i.e., checking in, observation of parent-child activity, explanation and coaching of new topic, setting goals, closing) and coaching characteristics (i.e., collaborative, reflective, nonjudgmental, performance-based) for the therapist to follow in each telehealth session. The measures also followed the ethical and practice guidelines of computer-mediated intervention including online communication, nonverbal feedback, and privacy and security issues [39]. 

These areas were developed into a five-point Likert-based fidelity rating system (separate from the fidelity system used with parents) and practiced by the therapist in order to maintain 85% correct delivery across three consecutive telehealth sessions with two pilot families before enrolling participants in the current study. An independent, trained rater continued to ensure that the therapist maintained at least 85% correct delivery across approximately 75% of the sessions delivered. 

### 2.4. Study Design and Procedure

 A single-subject, multiple-baseline design was conducted across the nine parent-child dyads [40]. Dyads were randomly assigned to different pre-determined baseline periods ranging between 4 and 11 probes. During baseline, parents and the therapist video-conferenced twice per week to observe parent-child interactions inside play, meal, and/or caretaking activities at home. Approximately two 10-minute probes were collected at the start and end of each one hour session. Parents were encouraged to carry out the activity as they normally would do with their child in the comfort of their own home. There was no instruction or expectation of the “right way” to do an activity but rather to highlight positive or challenging behaviors and real home-life interactions to the therapist. Generally, parents selected children's highly-preferred toys or physical games (e.g., peek-a-boo, chase, tickle, spinning around the room) to engage their child in play and/or care-giving tasks (e.g., feeding, dressing, diapering, chores) to demonstrate children's level of independence within these routines. 

No coaching or information related to the intervention was provided during families' baseline period. Instead the therapist and parents completed the ESDM Curriculum Checklist [35], a 480-item assessment of child skills across areas of development (i.e., receptive and expressive communication, social interaction, imitation, cognition, joint attention, play, self-care, and fine and gross motor). Parents received hard copies of the Checklist (prior to the first preintervention session) and together with the therapist, marked the individual behaviors children demonstrated consistently and with ease versus behaviors that occurred infrequently or had yet to develop. When necessary, the therapist suggested activities, interactions, or strategies to help the parent evaluate specific skills. For example, the therapist might suggest that the parent model the skill a few times to see whether the child would naturally imitate the action before offering physical assistance. Overall, parents identified a set of 10–12 behaviors to teach their child (e.g., increasing verbalizations, responding to questions, playing with toys more appropriately) across the 12-week remote coaching program. 

#### 2.4.1. Intervention

Once parent-child dyads completed their specific baseline phase, they immediately received telehealth delivery of the intervention. The parenting intervention was the ESDM, an evidence-based approach for stimulating developmental growth in young children with ASD [11]. The ESDM aims to create an affectively warm and rich teaching environment to foster positive relationships between children and their social partners. The model approaches language development from a communication science orientation, emphasizing the social function of language and the development of nonverbal communication and imitation as foundations for verbal language. A detailed parent manual highlights 10 therapy strategies [9] related to: (a) increasing the child's attention and motivation; (b) sensory social routines; (c) dyadic engagement; (d) non-verbal communication; (e) imitation; (f) antecedent-behavior-consequence relationship (ABCs of learning); (g) joint attention; (h) functional play; (i) symbolic play; (j) speech development. 

**Figure 1:**
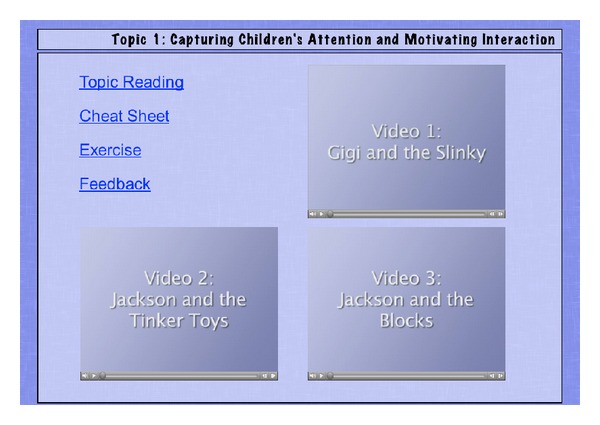
Screen shot of topic one from the ESDM learning module DVD.

In the Vismara et al. study [6], parents received in-print weekly readings, learning activities, and self-evaluations related to the selected ESDM topic in order to teach specific skills to their child within daily interactions at home. In this study, all intervention materials were transferred to a DVD with the addition of video recorded examples of the therapist demonstrating each ESDM topic with children of different ages, skill level, and ethnicity. The parent clicked an audio-recorded narration before or after selecting the video to explain the key strategies and child behaviors illustrated in each recorded activity (see Figure 1 for screen shot). Next to each online topic were links to recommended activities for parents to try with their child at home, as well as placeholders for parents to electronically enter their feedback and questions and discuss with the therapist during the video-conferencing sessions. Parents were mailed the DVD following their completion of the baseline phase and instructed to read the topic and watch the related videos in preparation for their upcoming session. Each module took approximately 20 minutes to view and parents followed the order of topics listed above since understanding of the earlier content was crucial for the implementation of the later topics. Parents were also able to revisit earlier modules either for their own instruction or with the therapist for additional review at any point during the program.

For 12 weekly one-hour sessions, the therapist and parent logged onto a secure software program to initiate the video conferencing call, allowing each other to see, hear, and communicate in real time. At the start of the session, the parents' progress from the past topic was reviewed for 5–10 minutes, followed by a 10-minute parent-child play activity that provided data on child and parent behaviors of interest. The activity also allowed the therapist to observe the parent's skill delivery from the prior week's topic and if needed, to coach and strengthen technique use during additional home activities (via video conferencing) before proceeding to the next topic. The therapist then verbally discussed the next topic with parent feedback and examples about the relevance and appropriateness of the reading and video materials to the child's learning needs, as well as anticipated challenges to trying the activities at home with their child. 

Following discussion, the parent practiced the set of new strategies in the context of at least two play or caretaking activities in their home aimed at teaching child behaviors from their list of identified developmental goals. An example of this might include the parent applying the teaching strategies from the attention/motivation lesson such as optimizing face-to-face positioning, minimizing outside distractions, imitating the child's play actions within a drawing activity at the kitchen table to teach the following developmental behaviors: (a) expressive communication (e.g., consonant-vowel speech production when asking for a marker, piece of paper, or a certain picture to be drawn); (b) receptive communication (e.g., looking to the parent when called by name before receiving a requested material or following an instruction to put the cap on the marker before receiving the next one or to help draw a certain shape before continuing the activity); (d) joint attention (e.g., pointing to or holding up the picture out of interest to share with the parent); (e) cognition (e.g., labeling the marker's color, counting the number of pictures drawn on the paper, or tracing letters); (f) fine motor (e.g., using an appropriate grip to hold the crayon or marker); (g) social interaction (e.g., drawing shapes on the paper); (h) social interaction (e.g., taking turns with the parent to draw a picture); (i) and/or imitation (e.g., copying the parent's motions drawn on paper). 

The therapist's coaching style adopted adult learning principles to facilitate parents' acquisition of the topic content, including joint planning, observation, active listening, as well as reflective questioning to encourage parent evaluation about the strategies practiced and what to try next [38]. For example, if the parent was struggling with the dyadic engagement topic on how to build a joint activity with their child, the therapist might begin the conversation by asking, “What was your goal for this activity?” (i.e., joint planning), in which case the parent might respond that she wanted her son to stay seated and draw with her at the table. The therapist could then ask, “What worked and did not work well in that activity?,” to which the parent could answer that having the child choose what color crayon and animal to draw on the paper kept him engaged longer in the activity versus making those decisions for him. Next, the therapist might ask, “Now thinking about what your son likes and does not like about drawing, what would you try differently the next time the two of you attempt this activity?” to which the parent might identify more choice-making opportunities to offer her son in the subsequent activity. If parent fidelity did not improve within two consecutive sessions, the therapist used progressively more directive coaching practices to improve parent performance, such as specific questions (e.g., “I wondered what would happen if you tried X”), suggestions (e.g., “Remember that when you do X, your child will likely do Y”), role play and lastly modeling the skill.

During the final 10–15 minutes of each session, the therapist and parent identified at least two different natural routines each day at home to continue practicing the techniques (e.g., playing with toys and games, eating meals, reading books, household tasks, face-to-face games without objects). The parent formulated an action plan of daily times or activities for when specific teaching topics and child objectives could be embedded within home routines. It was not expected that parents maintain data of child skills unless they specifically asked to be taught this component of the intervention; otherwise the therapist recorded weekly child progress across the individual teaching activities conducted by parents. 

#### 2.4.2. Follow-Up

After 12 continuous weeks of intervention, parents and their children connected to the video-conferencing program for three additional one-hour sessions, each scheduled two weeks part, to assess maintenance of ESDM skill delivery. A 10-minute parent-child play activity was recorded at the start of each session, followed by parent updates of skill use and child progress and coaching if necessary to address treatment drift in techniques during the final half of the session.

### 2.5. Dependent Measures

The first 10 minutes of a parent-child activity completed at the start of each session was videotaped from the video-conferencing program for later scoring of child language, imitation, social engagement, and parent fidelity of implementation and interactive behavior. Session data were reported across the baseline, 12-week intervention, and 6-week follow-up period for each parent-child dyad. Research assistants trained by the first author served as primary coders with the first author conducting reliability checks on all measures. The assistants were undergraduate students in psychology and had been volunteering at our center and trained to agreement with the first author for one year prior to their involvement in this study. Reliability training involved careful reading of the operational definitions of the dependent variables and practice in rating the responses of the children and parents. The coders were blind to the study's hypotheses and scored videotaped probes in random order to minimize expectations regarding child and parent progress. For each dependent variable, inter-rater agreement was established prior to scoring and maintained throughout the study by having assistants independently rate and compare 33% the observations.

#### 2.5.1. Fidelity of Implementation

 The ESDM Fidelity Scale [35] evaluated parents' use of 13 interactive behaviors on a 5-point Likert-based scale from a 1 or no competent teaching to a score of 5 or extremely competent teaching. Fidelity defined by a total score of 80% or scores of 4 or greater taught concepts such management of child attention, motivation, and arousal needs, in addition to sensitivity, responsivity, and expansion of child nonverbal and communicative behaviors. Inter-rater agreement was defined as coders' scores falling within one point on the Likert scale for each item. Agreement was 97%, with a kappa of 0.97.

#### 2.5.2. Child Social Communication Behaviors

Videotaped sessions were transcribed and scored for child production of spontaneous and prompted functional verbal utterances including single words or approximations and imitative play actions on objects and gestures (see [6] for detailed definitions). Raters who were blind to the time point coded child behaviors. Examination of the data revealed very few instances of prompted imitation and thus only spontaneous imitation was used. The overall ICC between two independent coders blind to the time point was 0.84, with a range of 0.70–1.00 for all types of language and 0.88 with a range of 0.75–1.00 for imitative behaviors.

#### 2.5.3. Observation Ratings of Parent and Child Engagement

The Maternal Behavior Rating Scale (MBRS [41]) and Child Behavior Rating Scale (CBRS [42]) were used to assess parents' interaction styles and children's engagement, respectively, across a five-point Likert rating scale ranging from 1 (very low) to 5 (very high). The MBRS characterizes the parent's style of relating to or caring for the child across four categories ranging from levels of responsiveness and sensitivity to the child's overt and subtle needs, to enjoyment and warmth displayed during the interaction, and to amount of encouragement, directiveness and teaching pace for helping children accomplish tasks. For the CBRS, the child is evaluated across two categories, engagement and interest in the activity, as well as joint attention, creativity, and affect demonstrated toward the parent. For Likert-scale ratings of both measures, an agreement was defined as both observers giving the exact rating on a probe-by-probe basis. Agreement was 80% with a kappa of 0.79.

#### 2.5.4. Feasibility and Acceptability Questionnaire

 At the end of the 12-week intervention, parents completed a eight-item questionnaire about: (a) their concerns using technology to help their child learn prior to starting the Internet-based coaching program and their perceptions following the experience; (b) the individual, familial, technological, and/or ecological barriers to completing the program; (c) their perceptions of the coaching style and expectations for parent-child change; (d) the most and least helpful aspects of the program. 

### 2.6. Analytic Plan

 For data collected weekly, such as behavioral observations of child behaviors, parent fidelity, and Mahoney ratings of child and parent behaviors, analyses were first conducted on weekly baseline data to assess whether any change was occurring in the absence of treatment (i.e., whether the baseline measures were stable). This was followed by analysis of change in behaviors over time during the treatment session in order to assess whether behavior changes occurred specifically during the treatment phase. For standardized tests (i.e., the MacArthur CDI and Vineland), analyses were conducted on change from baseline to follow-up. All such repeated measures data were analyzed using generalized estimating equations (GEE), yielding Wald chi-square tests of model effects (e.g., time). To assess correlations between variables over time, standardized regression coefficients were calculated from single predictor longitudinal regression models using z-scores.

## 3. Results

Table 2 shows the data for all dependent variables discussed below at both baseline and at the 18-week follow-up phase.

### 3.1. Parent Fidelity

**Figure 2:**
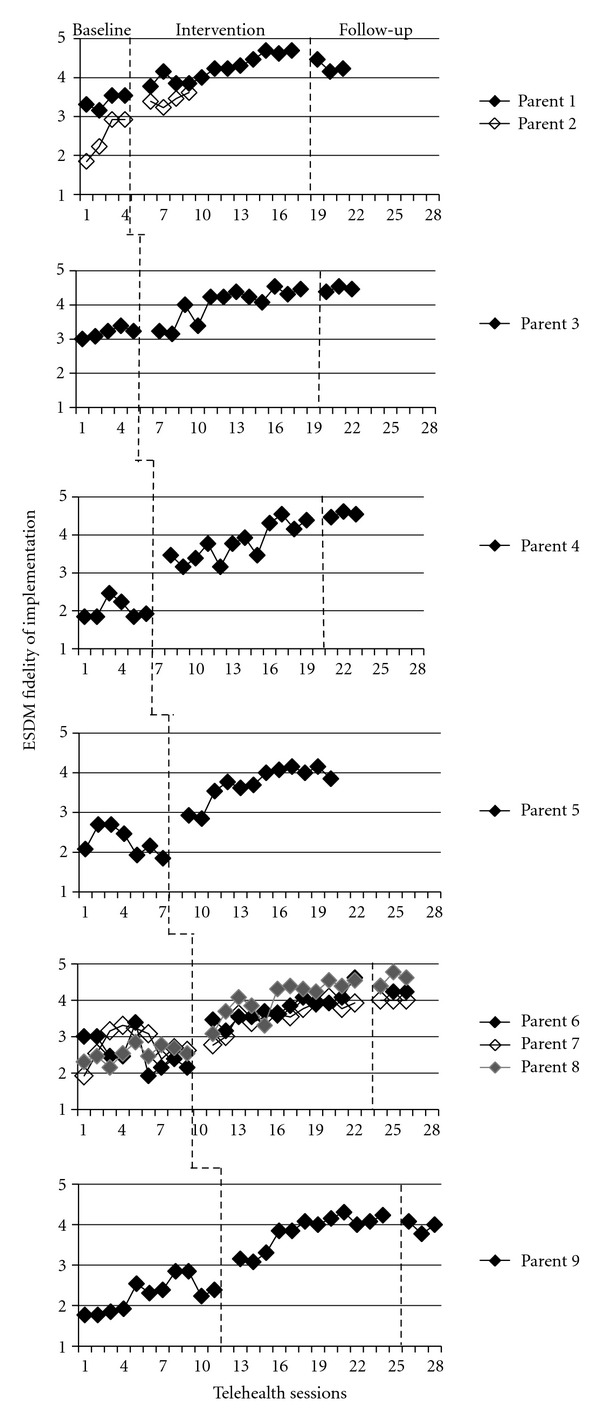
Individual parent ESDM fidelity scores.

 Analysis of parent fidelity ratings during baseline revealed no significant change over time (*X* ^2^ = 1.44, df = 1, *P* = .23) and an overall average at baseline of 2.62 (SD = 0.44)—well below the target fidelity of 4.00. In contrast, analysis of parent fidelity over time during treatment revealed significant increases (*X* ^2^ = 342.58, df = 1, *P* < .001, *d* = 4.62). The average time to achieving fidelity (at or above an average rating of 4.00) was 6.41 weeks (SD = 4.35). Individual data points are shown in Figure 2 with parents receiving the same number of baseline probes grouped together in the same tier. 

### 3.2. Mahoney Ratings of Parent and Child Engagement

Mahoney scale ratings for 4 domains of parent behavior, measured each week, were moderately correlated with each other, ranging from *r* = .80 for parental responsivity and affect to *r* = .17 for parental responsivity and directive behavior. There were no significant changes in ratings of parental behavior over time during baseline. During treatment, significant increases in parental behavior ratings from baseline to follow-up were seen in responsivity (*χ* ^2^ = 77.31, df = 1, *P* < .001, *d* = 2.41), affect (*χ* ^2^ = 60.42, df = 1, *P* < .001, *d* = 2.19), and achievement oriented behaviors (*χ* ^2^ = 40.25, df = 1, *P* < .001, *d* = 2.89). There was no significant change in parent directive behavior over time during intervention. 

The relationships between ESDM fidelity ratings and each of the first 3 Mahoney parent ratings over time were all high and positive, with predictive standardized coefficients (i.e., correlation) ranging from 0.78 for parental affect and fidelity to 0.80 for parental responsivity. In contrast, there was no relationship between parental directive behavior and fidelity over time, with a standardized regression coefficient of .15 (*P* = .17). 

The two Mahoney child scale ratings—attention and initiation—were highly correlated at *r* = .83. No significant changes in ratings of child behavior were seen during baseline. Significant increases were seen from baseline to follow-up for both child attention (*χ* ^2^ = 158.42, df = 1, *P* < .001, *d* = 3.94) and child initiation (*χ* ^2^ = 145.34, df = 1, *P* < .001, *d* = 3.11). Standardized coefficients between both child rating variables and parental ratings of responsivity, affect, and achievement over time were all significant and highly positive (from *r* = 0.64 to 0.82), suggesting that child behaviors increased over time in concert with parental behaviors. Mahoney ratings of child behavior were also moderately correlated with child behavioral observation measures of words and imitation over time, ranging from *r* = 0.51 to *r* = 0.68.

### 3.3. Child Social-Communication Behaviors

**Figure 3:**
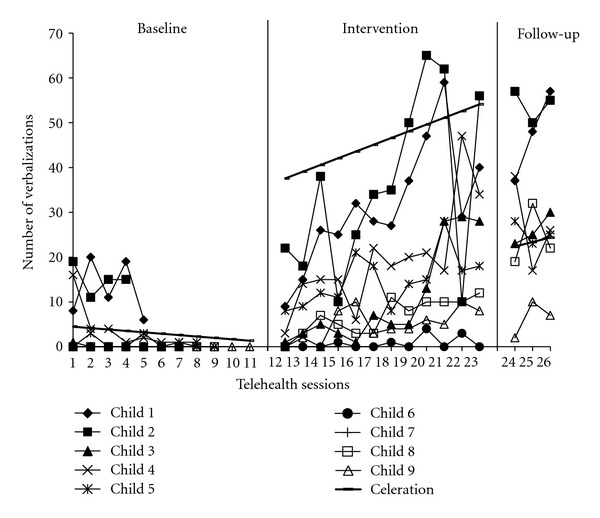
Individual child spontaneous language.

**Figure 4:**
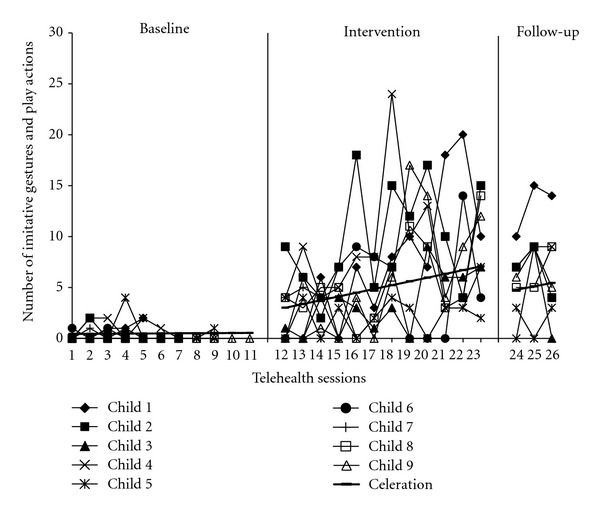
Individual child spontaneous imitative behaviors.

 Analyses of child behaviors during baseline sessions revealed no increases over time. Analyses of weekly change during treatment revealed significant increases for all behaviors over time. For spontaneous functional verbal utterances, there was a significant overall increase (*χ* ^2^ = 103.93, df = 1, *P* < .001, *d* = 2.20) over time. There was also a significant increase in prompted words over time (*χ* ^2^ = 30.03, df = 1, *P* < .001, *d* = 1.58). The correlation between spontaneous and prompted words was high (*r* = .70), and, not surprisingly, there was thus a significant increase for total words combined over time as well (*χ* ^2^ = 131.66, df = 1, *P* < .001). Analysis of observed instances of spontaneous imitation also revealed a significant effect for time (*χ* ^2^ = 27.66, df = 1, *P* < .001, *d* = 2.59). Individual linear trajectories of child spontaneous language and imitative behaviors are shown in Figures 3 and 4, respectively. Children received the same number of baseline probes as their parents; however, data were pooled together to highlight group trends over time superimposed in bold.

### 3.4. Standardized Testing Data

Parallel to the observed number of words produced weekly in each session over time, analysis of the MacArthur vocabulary production at baseline and at follow-up revealed a significant overall increase (*χ* ^2^ = 24.69, df = 1, *P* < .001, *d* = 1.63). An analysis of the MacArthur vocabulary comprehension also revealed a significant increase over time (*χ* ^2^ = 20.57, df = 1, *P* < .001, *d* = 1.60).

Analysis of the Vineland revealed a significant effect for time on the adaptive behavior composite (*χ* ^2^ = 5.69, df = 1, *P* < .05, *d* = 0.40). Nevertheless, inspection of individual data points revealed that this modest overall increase appeared due primarily to two initially low-scoring subjects making large gains by follow-up and thus this result may best be characterized as regression to the mean.

### 3.5. Feasibility and Acceptability Questionnaire

All nine parents answered the questionnaire's open-ended questions soliciting feedback about the Internet-based coaching program. Research assistants independently reviewed and categorized parents' responses into themes described below. Eight out of the nine parents expressed initial concerns about whether telehealth delivery would provide enough support to change behavior and the logistics of using the software program, whereas one parent had no concerns prior to starting the study; however, when asked whether initial concerns were addressed by the end of the study, all eight parents felt reassured and perceived the distance coaching as informative and as valuable as live in-home or center-based sessions delivered by professionals. Six parents identified the video examples on the DVD as more useful teaching aids than the reading handouts; whereas the other three parents noted that weekly video conferencing plus DVD learning model was interactive, helpful, and easier to use than first anticipated. All parents described some degree of frustration when using the video conferencing program, such as the Internet connection freezing in mid-conversation or the audio or web-camera not working when first connecting with the therapist; however, they thought the therapist quickly helped them resolve the problems. Interestingly, parents did not mention having to move the laptop or web-camera (if not built into the laptop) around to capture footage as a limitation. Rather, they noted difficulty with siblings wanting to participate in the sessions or their work schedules limiting time spent with their children. Finally, all parents agreed that they would recommend this approach to other parents of children with ASD, particularly when community services are scarce and/or confronted with long wait lists.

## 4. Discussion

Parent-implemented interventions are designed to help families create teachable moments each and every day to promote their child's development [43]. If parents cannot access the intervention regularly or find the intervention too difficult to implement throughout daily life, parents likely will not use the techniques when on their own, let alone achieve high fidelity of implementation [13]. Today's technology may not only help professionals reach out to a greater number of families with limited community-based resources but also help the intervention fit better within families' lifestyles and routines to promote more active and meaningful learning. 

The primary goal of this study was to determine whether telehealth delivery of a parenting intervention program would support parent-child learning throughout regular activities at home. Nine families with ASD received a DVD learning module and 12 weeks of one-hour live streaming video conferencing sessions in the ESDM. The remote coaching allowed parents to share a range of interactions, locations, and child behaviors for therapist feedback. Parents achieved fidelity in the ESDM within six weeks of starting the intervention and maintained gains across the six weeks of follow-up. This finding was similar to Vismara et al. [6], which demonstrated that parents approached fidelity approximately halfway through the center-based program and maintained their skills throughout the same follow-up period. The fact that parents were successful in learning similar content at a distance may support the use of telehealth for making services more accessible to families without necessarily compromising the quality of intervention to be taught. 

As parents' fidelity improved, their interaction styles also changed with increased ratings of attentiveness, responsiveness, sensitivity, and enthusiasm to their children's needs and communicative behaviors. It was encouraging that telehealth did not disrupt or interfere with parents' style of relating to or caring for their children and supports earlier work that children's developmental outcomes are closely tied to parenting behaviors [41]. Parents also reported high satisfaction with the DVD learning module and video-conferencing, describing both features as dynamic, easy to use, and supportive even without a therapist's physical presence. They valued the opportunity to share everyday, realistic moments with the therapist throughout their home and in doing so, felt more learning opportunities and behaviors could be targeted during intervention. 

 Across the intervention period, children's social-communicative behaviors increased significantly as evidenced by three independent data sources. First, children initiated novel, meaningful, and pragmatically-appropriate language throughout typical activities at home with their parents. Their language did not remain static or dependent upon adult cues but matured into spontaneous and independent speech across intervention and follow-up. Similar rates of acquisition were noted for children's use of imitative play actions and gestures inside rich, context-dependent interactions at home with parents. 

Second, weekly ratings of children's joint engagement, social interest, and shared positive affect increased over time in concert with parental behaviors. Children appeared to relate to their parents as social partners, constructing mutually enjoyable activities rather than maintaining interest only in the objects used during play. These ratings appeared moderately correlated with the gains in words and imitation over time, supporting parents' ability to create teachable moments at home. 

Finally, parents reported substantial growth in their children's development, noting comprehension and use of language and gestures as the primary gains. It is important to mention that all child changes occurred relative to stable baseline measures of behavior over time and without direct intervention or coaching from the therapist suggests the feasibility of telehealth delivery for improving parent and child behaviors in a short period of time. 

The results of this study are preliminary and thus should be interpreted cautiously given the methodological limitations. Although the use of single-subject methodology allows for a detailed examination of program feasibility and efficacy, it is unknown how well the results from the current study would generalize to other families. The sample size was very small and relatively homogeneous, consisting primarily of Caucasian, middle-class families, all highly motivated and with the equipment including laptops, web-cameras, and Internet access necessary for participation, although equipment was available to families in need. It is unclear how this approach would work for families that have limited access to these resources and whether effective learning could take place in other public settings, such as libraries, community agencies, hospitals, or schools. Additionally, parent improvement was primarily assessed with a global rating scale rather than evaluating individual areas of change, making it difficult to pinpoint exact constructs of behavior that may mediate better or poorer treatment outcomes. 

In terms of child change, weekly ratings were collected at the start of each in-home session before any coaching or instruction occurred; however, the probes were not standardized in terms of the play materials used between parents and children. Instead the selected activities represented whatever real-life moments parents had been experiencing with their children during the given week and thus it is possible that parents could have selected those materials and activities most motivating to increase children's cooperation. This and other standardization issues related to equipment use (e.g., second adult operating the laptop and web-camera) are important to specify for subsequent efficacy trials attempting to replicate findings. Finally, the use of only one therapist to provide telehealth delivery was necessary due to limited funding for this study; however, it questions how easily the approach would transfer to other therapists and/or community practices. 

Future studies will need to recruit a larger and more diverse sample to examine the use of telehealth with participants of different ethnicities, levels of education, and income. It will also be important to standardize the use of equipment and training protocols for effectiveness trials in community-based health settings. An important part to this dissemination will be in helping providers develop “telecompetence” [17] in both equipment use, such as how to maintain reliable broadband connections with families, and in professional development areas such as communicative strategies to deliver and receive information effectively, interpersonal skills to develop the rapport and trust with families, and methods for protecting families' rights to privacy via the Internet [44–46]. The current study provided a framework for developing and testing a telehealth approach to service families; however, the preliminary nature of the findings makes it impossible to confirm its efficacy let alone its effectiveness in comparison with other community practices. Additional research is currently underway in our center to continue testing and strengthening our approach to telehealth-delivered parent intervention with the goal of disseminating a defined parent training curriculum to community early intervention programs.

Recent trends with large, randomized and methodologically sound parent-implemented studies note strong effects for assessor-rated parent-child interactions and yet such gains have not produced a “downstream” effect on ASD symptoms, particularly when compared to community programs ([7, 8, 10]; Carter et al., 2011). Some have suggested that perhaps the standard of community care is higher than originally anticipated (e.g., [8]), standardized outcome measures such as the ADOS or ADOS-G may lack sensitivity as a measure of change [7], or that the quality, quantity, consistency, and generalization of parent intervention usage may be more difficult than expected for parents to do in daily life [13]. An important challenge then is how to strengthen the effects of parent mediated interventions.

The current study represents an innovative approach to helping parents make the most out of learning inside daily play and caretaking activities at home without increasing the cost of service. We are continuing to test additional telehealth modalities that might make complex intervention strategies easier, more efficient, and affordable to use with parents and their children with ASD. One notion is that with 77% of US families now having Internet access (International Telecommunication Union, 2011— http://www.itu.int/en/about/Pages/overview.aspx/), we are developing simple intervention aides that parents can access via online modalities to easily record and view children's progress across learning goals, as well as specify the amount and type of teaching opportunities delivered throughout daily routines at home and in the community. Parents can then electronically send this information to therapists and by doing so, generate a clearer picture of intervention intensity delivered in natural routines based upon research standards (i.e., dose, form, frequency, duration; [47]). 

The potential of telehealth-delivered intervention to equip families with timely and comprehensive services is great. Our hope is that telehealth will enhance parent-delivered intervention by offering parents more choice in what skills they want their children to learn, when and where they want to teach them, and how such interactions can translate into observable achievements to motivate their continued efforts in helping their children develop and grow. Although the study offers small, preliminary support at best, we are optimistic that such technology presents advantages to accessing more families and making interventions easier to use in the context of daily life. 

## Figures and Tables

**Table 1:** Baseline standardized test results.

Variable	*N*	Mean	SD	Min	Max
Age at pre-treatment (in months)	9	28.89	7.64	16	38
Treatment length (in days)	9	93.78	27.85	32	134

Mullen visual reception *t*-score	9	33.78	13.96	20	65
Mullen fine motor *t*-score	9	33.00	17.60	20	66
Mullen receptive language *t*-score	9	28.78	10.91	20	46
Mullen expressive language *t*-score	9	27.44	9.62	20	42
Mullen visual reception age equivalent	9	17.22	8.91	1	33
Mullen fine motor age equivalent	9	19.67	5.98	12	31
Mullen receptive language age equivalent	9	13.78	6.40	7	24
Mullen expressive language age equivalent	9	13.78	6.51	5	23
Mullen early learning composite	9	70.44	30.16	49	140

Vineland communication standard score	6	73.67	13.14	61	91
Vineland daily living standard score	6	82.33	11.45	71	102
Vineland socialization standard score	6	78.33	14.51	61	100
Vineland motor standard score	6	86.00	10.00	75	100
Vineland adaptive behavior composite	6	77.00	12.39	65	97

ADOS communication + social total	9	17.44	3.88	10	23

**Table 2:** Baseline and follow-up measures of change.

Measure	Baseline	Follow-up
Parent fidelity^a^	2.62 (.44)	4.29 (.26)
MBRS parent responsivity^a^	2.74 (.61)	4.14 (.55)
MBRS parent affect^a^	2.74 (.52)	3.99 (.62)
MBRS achievement oriented behaviors^a^	2.46 (.48)	3.79 (.44)
MBRS directive behavior^a^	2.94 (.65)	3.26 (.41)
CBRS attention^a^	2.46 (.51)	3.92 (.12)
CBRS initiation^a^	2.15 (.50)	3.56 (.40)
Spontaneous verbalizations^b ^	3.44 (5.79)	29.86 (15.95)
Prompted verbalizations^b^	1.89 (2.71)	14.57 (11.03)
Spontaneous imitation^b^	0.44 (.53)	6.57 (3.31)
MacArthur CDI vocabulary^c^	39.71 (39.87)	147.43 (84.55)
MacArthur CDI comprehension^c^	107.57 (66.44)	237.15 (93.14)
Vineland^c^	77.00 (12.39)	81.29 (9.01)

^a^Refers to a five-point Likert-based rating scale; ^b^refers to frequency scores; ^c^refers to standard scores.

## References

[1] Bruner J (1981). The social context of language acquisition. *Language and Communication*.

[2] Stahmer AC, Gist K (2001). The effects of an accelerated parent education program on technique mastery and child outcome. *Journal of Positive Behavior Interventions*.

[3] Coolican J, Smith IM, Bryson SE (2010). Brief parent training in pivotal response treatment for preschoolers with autism. *Journal of Child Psychology and Psychiatry and Allied Disciplines*.

[4] Ingersoll B, Gergans S (2007). The effect of a parent-implemented imitation intervention on spontaneous imitation skills in young children with autism. *Research in Developmental Disabilities*.

[5] Rogers SJ, Hayden D, Hepburn S, Charlifue-Smith R, Hall T, Hayes A (2006). Teaching young nonverbal children with autism useful speech: a pilot study of the Denver model and PROMPT interventions. *Journal of Autism and Developmental Disorders*.

[6] Vismara LA, Colombi C, Rogers SJ (2009). Can one hour per week of therapy lead to lasting changes in young children with autism?. *Autism*.

[7] Green J, Charman T, McConachie H (2010). Parent-mediated communication-focused treatment in children with autism (PACT): a randomised controlled trial. *The Lancet*.

[8] Oosterling I, Visser J, Swinkels S (2010). Randomized controlled trial of the focus parent training for toddlers with autism: 1-year outcome. *Journal of Autism and Developmental Disorders*.

[9] Rogers SJ, Dawson G, Vismara LA (2012). *An Early Start for Your Child with Autism: Using Everyday Activities to Help Kids Connect, Communicate, and Learn. Proven Methods Based on the Breakthrough Early Start Denver Model*.

[10] Aldred C, Green J, Adams C (2004). A new social communication intervention for children with autism: pilot randomised controlled treatment study suggesting effectiveness. *Journal of Child Psychology and Psychiatry and Allied Disciplines*.

[11] Dawson G, Rogers S, Munson J (2010). Randomized, controlled trial of an intervention for toddlers with autism: the early start Denver model. *Pediatrics*.

[12] Drew A, Baird G, Baron-Cohen S (2002). A pilot randomised control trial of a parent training intervention for pre-school children with autism: preliminary findings and methodological challenges. *European Child and Adolescent Psychiatry*.

[13] Kasari C, Gulsrud AC, Wong C, Kwon S, Locke J (2010). Randomized controlled caregiver mediated joint engagement intervention for toddlers with autism. *Journal of Autism and Developmental Disorders*.

[14] Koegel RL, Bimbela A, Schreibman L (1996). Collateral effects of parent training on family interactions. *Journal of Autism and Developmental Disorders*.

[15] Goodwin MS (2008). Enhancing and accelerating the pace of autism research and treatment. *Focus on Autism and Other Developmental Disabilities*.

[16] Dudding CC (2009). Digital videoconferencing: applications across the disciplines. *Communication Disorders Quarterly*.

[17] Turner JW, Thompson TL, Dorsey AD, Miller KI, Parrott R (2003). Telemedicine: expanding health care into virtual environments. *Handbook of Health Communication*.

[18] Baggett KM, Davis B, Feil EG (2010). Technologies for expanding the reach of evidence-based interventions: preliminary results for promoting social-emotional development in early childhood. *Topics in Early Childhood Special Education*.

[19] Boisvert M, Lang R, Andrianopoulos M, Boscardin ML (2010). Telepractice in the assessment and treatment of individuals with autism spectrum disorders: a systematic review. *Developmental Neurorehabilitation*.

[20] Wainer AL, Ingersoll BR (2011). The use of innovative computer technology for teaching social communication to individuals with autism spectrum disorders. *Research in Autism Spectrum Disorders*.

[21] Mitchell P, Parsons S, Leonard A (2007). Using virtual environments for teaching social understanding to 6 adolescents with autistic spectrum disorders. *Journal of Autism and Developmental Disorders*.

[22] Gibson JL, Pennington RC, Stenhoff DM, Hopper JS (2010). Using desktop videoconferencing to deliver interventions to a preschool student with autism. *Topics in Early Childhood Special Education*.

[23] Machalicek W, O’Reilly M, Chan JM (2009). Using videoconferencing to conduct functional analysis of challenging behavior and develop classroom behavioral support plans for students with autism. *Education and Training in Developmental Disabilities*.

[24] Hamad CD, Serna RW, Morrison L, Fleming R (2010). Extending the reach of early intervention training for practitioners: a preliminary investigation of an online curriculum for teaching behavioral intervention knowledge in autism to families and service providers. *Infants and Young Children*.

[25] Granpeesheh D, Tarbox J, Dixon DR, Peters CA, Thompson K, Kenzer A (2010). Evaluation of an eLearning tool for training behavioral therapists in academic knowledge of applied behavior analysis. *Research in Autism Spectrum Disorders*.

[26] Vismara LA, Young GS, Stahmer AC, Griffith EM, Rogers SJ (2009). Dissemination of evidence-based practice: can we train therapists from a distance?. *Journal of Autism and Developmental Disorders*.

[27] Feil EG, Baggett KM, Davis B (2008). Expanding the reach of preventive interventions: development of an internet-based training for parents of infants. *Child Maltreatment*.

[28] Kacir CD, Gordon DA (1999). Parenting adolescents wisely: the effectiveness of an interactive videodisk parent training program in Appalachia. *Child and Family Behavior Therapy*.

[29] MacKenzie EP, Hilgedick JM (1999). The Computer-Assisted Parenting Program (CAPP): the use of a computerized behavioral parent training program as an educational tool. *Child and Family Behavior Therapy*.

[30] Taylor TK, Webster-Stratton C, Feil EG, Broadbent B, Widdop CS, Severson HH (2008). Computer-based intervention with coaching: an example using the incredible years program. *Cognitive Behaviour Therapy*.

[31] Baharav E, Reiser C (2010). Using telepractice in parent training in early autism. *Telemedicine and e-Health*.

[32] Nefdt N, Koegel R, Singer G, Gerber M (2010). The use of a self-directed learning program to provide introductory training in pivotal response treatment to parents of children with autism. *Journal of Positive Behavior Interventions*.

[33] Jang, Dixon D J, Tarbox J, Granpeesheh D, Kornack J, de Nocker Y (2012). Randomized controlled trial of an eLearning program for training family members of children with autism in the principles and procedures of applied behavior analysis. *Research in Autism Spectrum Disorders*.

[34] Lord C, Rutter M, DiLavore PC, Risi S (1999). *The Autism Diagnostic Observation Schedule: The Manual*.

[35] Rogers SJ, Dawson G (2010). *The Early Start Denver Model for Young Children with Autism: Promoting Language, Learning, and Engagement*.

[36] Mullen EM (1995). *Mullen Scales of Early Learning*.

[37] Sparrow SS, Balla DA, Cicchetti DV (2005). *Vineland Adaptive Behavior Scales, Second Edition*.

[38] Hanft BE, Rush DD, Shelden ML (2004). *Coaching Families and Colleagues in Early Childhood*.

[39] Trepal H, Haberstroh S, Duffey T, Evans M (2007). Considerations and strategies for teaching online counseling skills: establishing relationships in cyberspace. *Counselor and Education*.

[40] Hersen M, Barlow DH (1976). *Single Case Experimental Designs: Strategies for Studying Behavior Change*.

[41] Mahoney G, Boyce G, Fewell RR, Spiker D, Wheeden CA (1998). The relationship of parent-child interaction to the effectiveness of early intervention services for at-risk children and children with disabilities. *Topics in Early Childhood Special Education*.

[42] Mahoney G, Wheeden CA (1998). Effects of teacher style on the engagement of preschool aged children with special learning needs. *Journal of Developmental and Learning Disorders*.

[43] National Research Council (2001). *Educating Children with Autism*.

[44] Socha McGee D, Cegala DJ (1998). Patient communication skills training for improved communication competence in the primary care medical consultation. *Journal of Applied Communication Research*.

[45] National board for Certified Counselors The practice of Internet counseling. http://www.nbcc.org/Assets/Ethics/internetCounseling.pdf.

[46] Srinivasan M, Keenan CR, Yager J (2006). Visualizing the future: technology competency development in clinical medicine, and implications for medical education. *Academic Psychiatry*.

[47] Warren SF, Fey ME, Yoder PJ (2007). Differential treatment intensity research: a missing link to creating optimally effective communication interventions. *Mental Retardation and Developmental Disabilities Research Reviews*.

